# Polymorphism in the IL4R gene and clinical features are associated with glioma prognosis: Analyses of case-cohort studies

**DOI:** 10.1097/MD.0000000000004231

**Published:** 2016-08-07

**Authors:** Tian bo Jin, Shuli Du, Xi kai Zhu, Gang Li, Yongri Ouyang, Na He, Zhiying Zhang, Yuan Zhang, Longli Kang, Dongya Yuan

**Affiliations:** a Key Laboratory for Molecular Genetic Mechanisms and Intervention Research on High Altitude Disease of Tibet Autonomous Region,School of Medicine, Xizang Minzu University, Xianyang, China; b Key Laboratory of High Altitude Environment and Genes Related to Diseases of Tibet Autonomous Region, School of Medicine,Xizang Minzu University, Xianyang, China; c Key Laboratory for Basic Life Science Research of Tibet Autonomous Region, School of Medicine, Xizang Minzu University, Xianyang, China; d School of Life Sciences, Northwest University, Xi’an, Shaanxi; e Department of Neurosurgery, Tangdu Hospital, The Fourth Military Medical University, Xi’an, China.

**Keywords:** glioma, *IL4R*, multivariate analysis, prognosis, univariate analysis

## Abstract

Inflammatory gene polymorphisms may be associated with glioma risk. The purpose of this study was to analyze effects of certain inflammatory gene and some clinical factors on patient survival.

The clinical information of 269 glioma patients conceived operation from September 2010 to May 2014 to decide the 1-, 3-year survival rates according to follow-up results and analyze age, gender, the WHO classification, extent of surgical resection, radiotherapy and chemotherapy factors effects on prognosis. Survival distributions were estimated by using the Kaplan–Meier method and difference in the survival was tested using the log-rank test. To estimate the association between the *IL4*, *IL13*, *IL10*, *IL4R* SNPs, and PFS and OS in glioma, the HR and 95% CI were calculated by univariate Cox proportional hazards model. Multivariate Cox model were performed to compute adjusted HR and 95% CI. All data was analyzed with SPSS17.0 package. Extent of surgical resection, chemotherapy, and age are an important factor in glioma overall survival and progression-free survival overall. Extent of surgery and chemotherapy are important factors in astrocytoma overall survival. Univariate analysis showed that *IL4R* rs1801275 was significantly associated with overall survival of glioma and astrocytoma patients (*P* < 0.05). Multivariate Cox regression analysis showed that *IL4R* rs1801275 GG genotype could increase the death risk of glioma and astrocytoma patients (Glioma: hazard ratio [HR]: 4.897, 95% confidence limits [95% CI]: 1.962–12.222, *P* = 0.001; Astrocytoma: HR: 15.944, 95% CI: 4.019–63.253, *P* < 0.05).

Our research results showed that extent of surgical resection, age, and chemotherapy affect the prognosis of glioma. The *IL4R* gene may affect the survival of glioma patients.

## 1 Introduction

Gliomas are highly malignant with poor prognosis.^[1]^ It is reported that the overall median survival in patients with glioma is ∼14.6 months with a 2-year survival rate at 30%.^[2]^ Glioblastoma (GBM), the most malignant brain tumor, has a 5-year survival rate of <10% and the median survival is 12 to 15 months.^[3]^ Histologic type, grade, age, extent of resection, tumor location, radiation therapy, some chemotherapy protocols, and Karnofsky performance status have close relationships with glioma survival.^[4]^ So far, there has been little progress made in improving treatment or patient survival.

To date, recent reports suggest that some gene polymorphisms can influence glioma survival, including *HLA-A*, *HLA-B*, *GLTSCR1*, *ERCC2*, *GSTP1,* and *GSTM1*.^[5–8]^ Scheurer ME et al. reported that the *IL4R* rs1805016 was significantly protective against mortality over time of glioma patients, and IL4R rs1805016 and rs1805015 TT genotypes were both significantly associated with survival beyond 1 year among high-grade glioma patients.^[9]^ These studies remind that genetic factors may be important in glioma prognosis.

Previous studies provide evidence that gene polymorphisms may play important roles in the prognosis of glioma in the European, whereas that is currently unknown in the Chinese population. Thus, the purpose of the current study was to examine the association between 7 common polymorphisms in the *IL4*, *IL13*, *IL10,* and *IL4R* genes and patient survival.

## 2 Materials and methods

### 2.1 Ethics statement

The use of human tissue and the protocol in this study were strictly conformed to the principles expressed in the Declaration of Helsinki and were approved by the Ethical Committee of Xizang Minzu University, Tangdu Hospital, the Fourth Military Medical University and Northwest University for approval of research involving human subjects. Signed informed consent was obtained from each participant.

### 2.2 Patient population and data collection

Gliomas patients (269) were randomly obtained at the Tangdu Hospital of The Fourth Military Medical University from September 2010 to May 2014. Histopathological diagnosis was confirmed by at least 2 senior neuropathologists according to the World Health Organization (WHO) classification.^[10]^ The WHO classification of tumors of the nervous system includes a grading scheme that is a “malignancy scale” ranging across a wide variety of neoplasms rather than a strict histological grading system. Grade I applies to lesions with low proliferative potential and the possibility of cure following surgical resection alone. Neoplasms designated grade II are generally in filtrative in nature and, despite low-level proliferative activity, often recur. Some type II tumors tend to progress to higher grades of malignancy, for example, low-grade diffuse astrocytomas that transform to anaplastic astrocytoma and glioblastoma. Similar transformation occurs in oligodendroglioma and oligoastrocytomas. The designation WHO grade III is generally reserved for lesions with histological evidence of malignancy, including nuclear atypia and brisk mitotic activity. The designation WHO grade IV is assigned to cytologically malignant, mitotically active, necrosis-prone neoplasms typically associated with rapid pre- and postoperative disease evolution and a fatal outcome. Widespread infiltration of surrounding tissue and a propensity for craniospinal dissemination characterize some grade IV neoplasms. This study was approved by the local ethics committee, and written informed consent was obtained from all patients.

Clinical information were collected and regularly updated for the patients with glioma through follow-up and questionnaires. These data include age, gender, date of diagnosis of primary tumor, date of surgical resection and surgery extent, treatment with chemotherapy and/or radiotherapy for primary and/or recurrent lesions, date of last follow-up, and status of patient (living/deceased) at the time of last follow-up. All the patients were followed-up every 2 months by telephone and deaths were recorded until May 2014.

### 2.3 Genotyping

Seven SNPs in the *IL4*, *IL13*, *IL10*, and *IL4R* genes were selected which previously reported to be associated with glioma, with minor allele frequencies >5% in the HapMap Chinese Han Beijing (CHB) population. DNA was extracted from whole-blood samples using the GoldMag-Mini Whole Blood Genomic DNA Purification Kit (GoldMag Co. Ltd. Xi’an City, China). DNA concentrations were measured with the NanoDrop 2000 (Thermo Scientific, Waltham, MA). Multiplexed SNP MassEXTENDED assay was designed by the Sequenom MassARRAY Assay Design 4.0 Software (Sequenom Co. Ltd., San Diego, CA).^[11]^ SNP genotyping with a standard protocol was performed using Sequenom MassARRAY RS1000 (Sequenom Inc., San Diego, CA).^[11]^ SequenomTyper 4.0 Software (Sequenom Inc., San Diego, CA) was used to analyze the data.^[11,12]^

### 2.4 Statistical analysis

Overall survival (OS) was calculated from the date of pathological diagnosis to the date of death or the last follow-up. PFS was calculated from the date of the pathologically confirmed to the progression of the disease, death without progression, or last clinical follow-up. We computed basic descriptive statistics for age at diagnosis, gender, WHO grade, extent of surgery, radiation therapy, chemotherapy, and frequencies of *IL4*, *IL13*, *IL10*, and *IL4R* variants. Survival distributions were estimated by using the Kaplan–Meier method and differences in the survival were tested using the log-rank test. To estimate the association of the *IL4, IL13*, *IL10*, and *IL4R* SNPs with PFS and OS in glioma, the HR and 95% CI were calculated by univariate Cox proportional hazards model. Multivariate Cox models were performed to compute HR and 95% CI, after adjustment potential risk factors. Statistical analysis was conducted using SPSS 17.0 statistical package (SPSS, Chicago, IL). All *P* values were 2 sided and *P* < 0.05 (2 sided) was considered statistically significant.

## 3 Results

Briefly, a total of 269 glioma patients were recruited, consisted of 145 males and 124 females, with mean age at 40.96 years. The median survival for the whole group was 11 months, and overall survival was 30.1% at 1 year and 6.5% at 3 years. The cases were categorized into147 WHO I-II and 122 WHO III-IV according to the World Health Organization (WHO) classification. All patients underwent tumor resection: 184 patients had total resection, and 85 patients had subtotal resection. In total, 245 patients (91.9%) were treated with radiotherapy, including 69 patients with conformal radiotherapy and 176 patients with gamma knife. Among all the 269 patients, there were 160 astrocytoma patients consisted of 88 males and 72 females, with mean of age was 42.8. The median survival for the astrocytoma group was 11 months, and overall survival was 28.8% at 1 year and 6.6% at 3 years. The distribution of the demographic characteristics, clinical variables, and genotypes studied were shown in Tables 1 and 2.

**Table 1:**
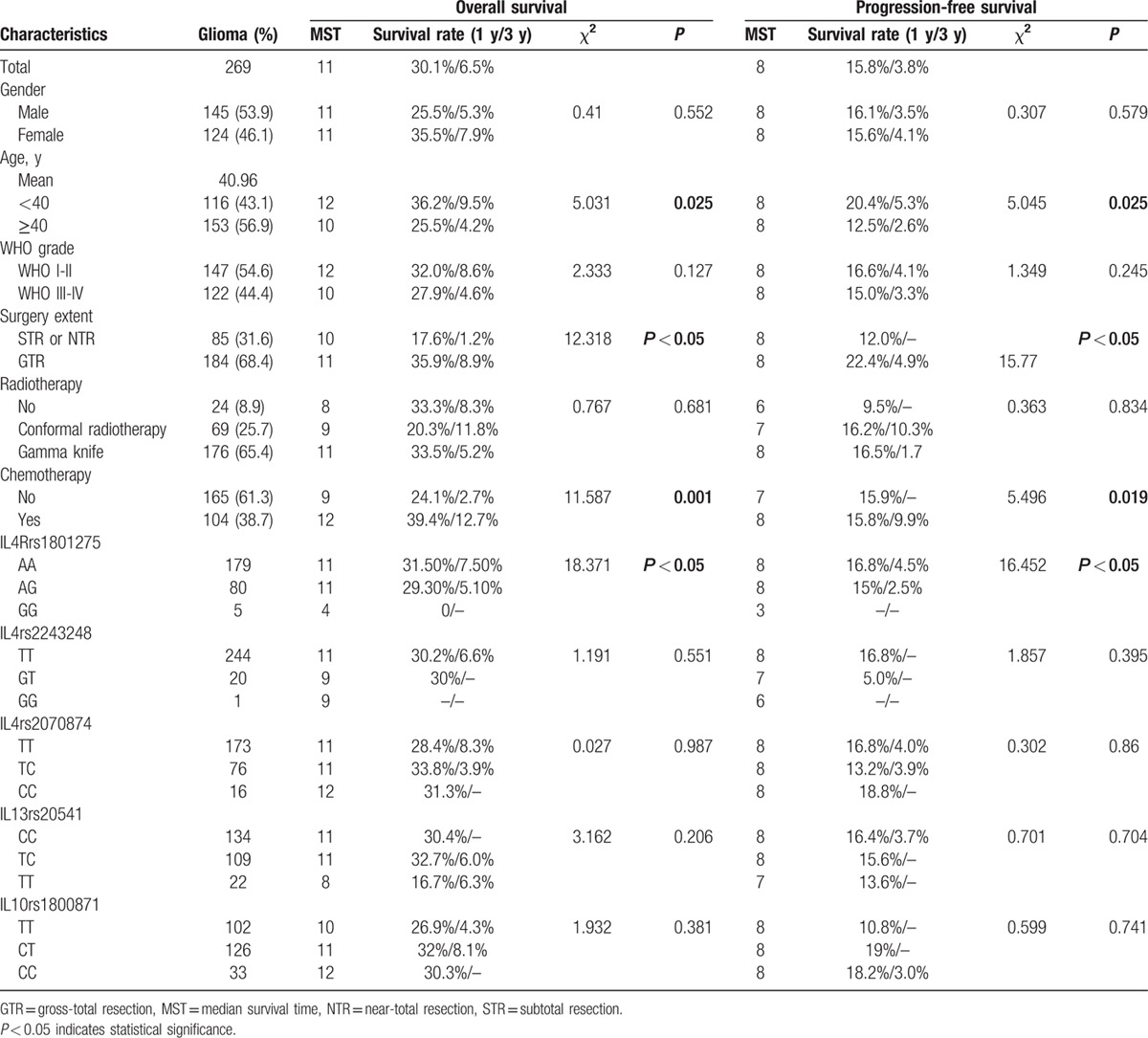
Glioma patient characteristics.

**Table 2:**
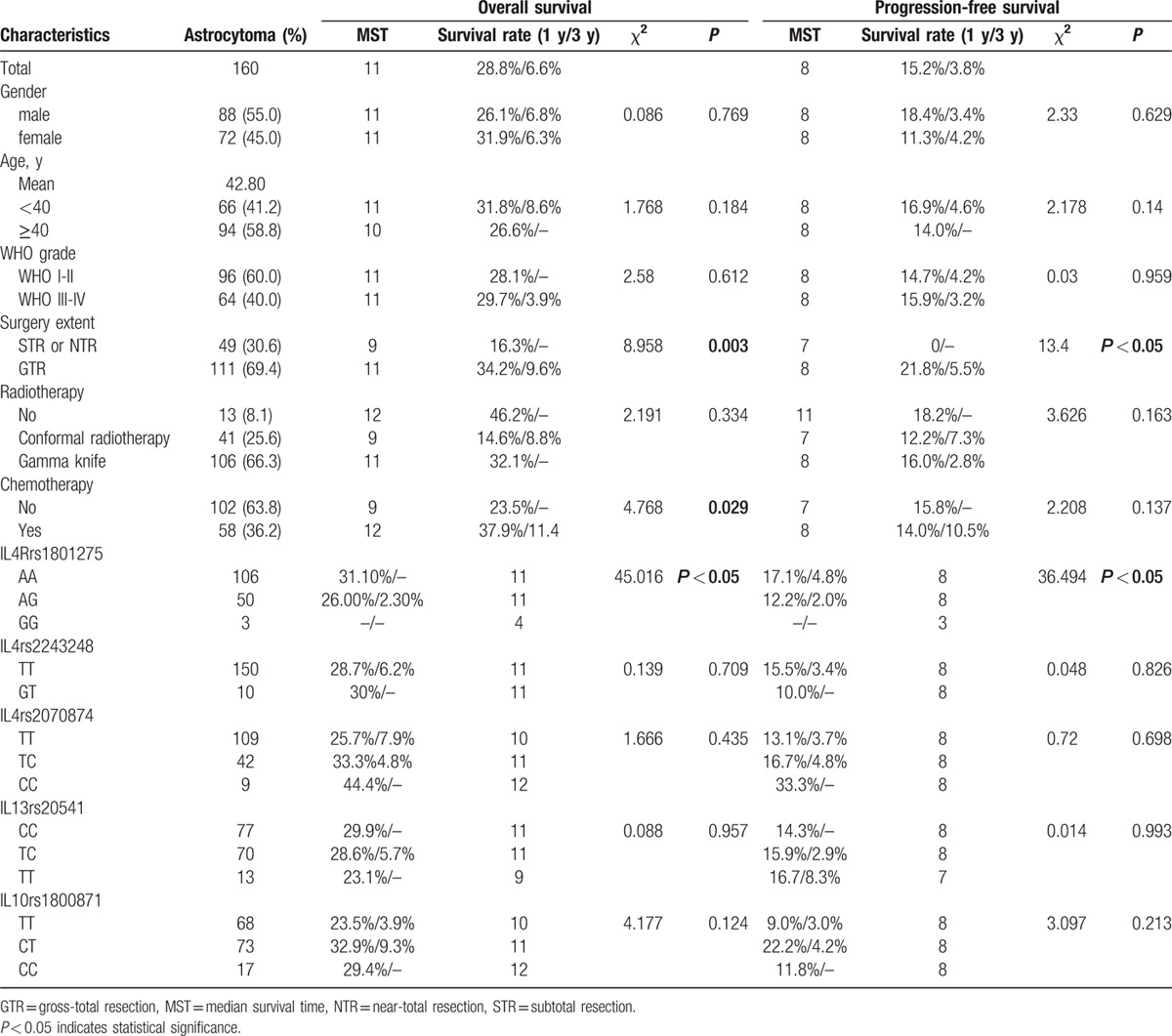
Astrocytoma patient characteristics.

A univariate analysis indicated worse prognosis in glioma patients with age at 40 or above (OS: *P* = 0.025; PFS: *P* = 0.035). Under the age of 40, the 1-year and 3-year survival rates of glioma patient were 36.2% and 9.5%, respectively; the 1-year and 3-year survival rates of the glioma patient aged 40 years or more were 25.5% and 4.2%, respectively (Tables 1 and 2).

A univariate analysis indicated better prognosis in glioma and astrocytoma patients with GTR (glioma: OS: *P* < 0.05, PFS: *P* < 0.05; astrocytoma: OS: *P* = 0.003, PFS: *P* < 0.05). The 1-year and 3-year survival rates of glioma patient with GTR were higher than those with STR or NTR, respectively. The 1-year and 3-year survival rates of glioma patient with GTR were 35.9% and 8.9%, respectively, whereas the STR or NTR glioma patient were 17.6% and 1.2%, respectively. Similar result was found in astrocytoma patient. The 1-year survival rate of astrocytoma patient with GTR was 34.2%, and the STR or NTR astrocytoma patient was 16.3% (Tables 1 and 2).

By the end of the follow-up, 104 patients with glioma showed responsive to chemotherapy, and 165 patients with glioma did not receive chemotherapy treatment. Better prognosis in glioma and astrocytoma patients with accept chemotherapy (glioma: OS: *P* = 0.001, PFS: *P* = 0.019; astrocytoma: OS: *P* = 0.029). The 1-year and 3-year survival rates of glioma patient with chemotherapy were 39.4% and 12.7%, respectively, which were higher than the patient without chemotherapy treatment (1-year survival rates: 39.4% vs 24.1%; 3-year survival rates: 12.7% vs 2.7%) (Tables 1 and 2).

**Figure 1:**
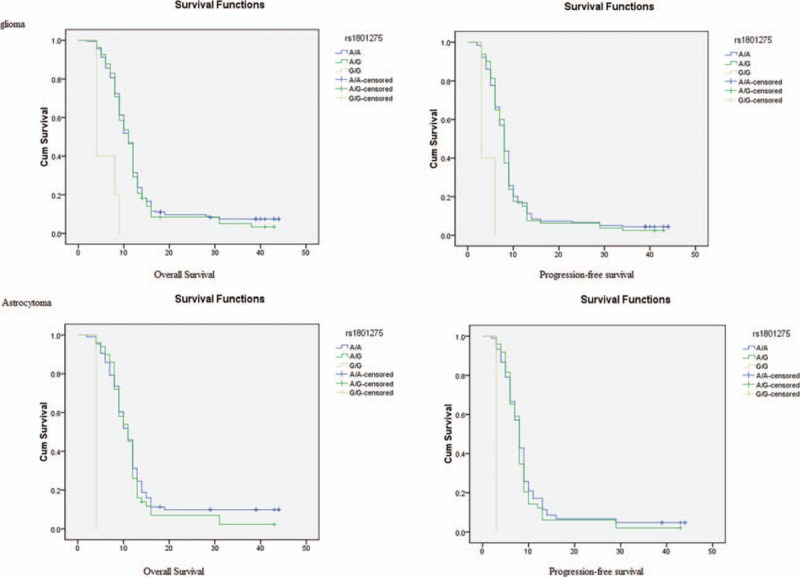
Kaplan–Meier analysis of overall survival and progressions-free survival are shown for different genotypes of *IL4R* rs1801275.

We examined the impact of the analyzed SNPs on the overall survival time of glioma and patients. The 1 year OS of glioma patients with *IL4R* rs1801275 GG (0%) and GA (29.3%) were lower than the AA (31.5%) genotype (*P* < 0.05). In this subgroup, astrocytoma patients with the AA, AG, and GG *IL4R* rs1801275 genotypes had 1-year OS rates of 31.0%, 26.0%, and 0%, respectively (*P* < 0.05) (Fig. 1).

The Cox proportional hazards model, adjusted for age, surgery extent, and chemotherapy, showed that patients carried 2 variant allele (GG) had significantly increased HR (HR, 4.897; 95% CI, 1.962–12.222; *P* = 0.001), whereas patients carried only 1 variant allele had a mildly significant HR (HR, 1.16; 95% CI, 0.883–0.526; *P* = 0.286). Using Cox regression, adjusted for surgery extent, and chemotherapy, we found that compared with astrocytoma patients carried AA genotype, subjects carried GG genotype had elevated risks of death with the HR (95% CI) of 15.944 (4.019–63.253) (Table 3).

**Table 3:**
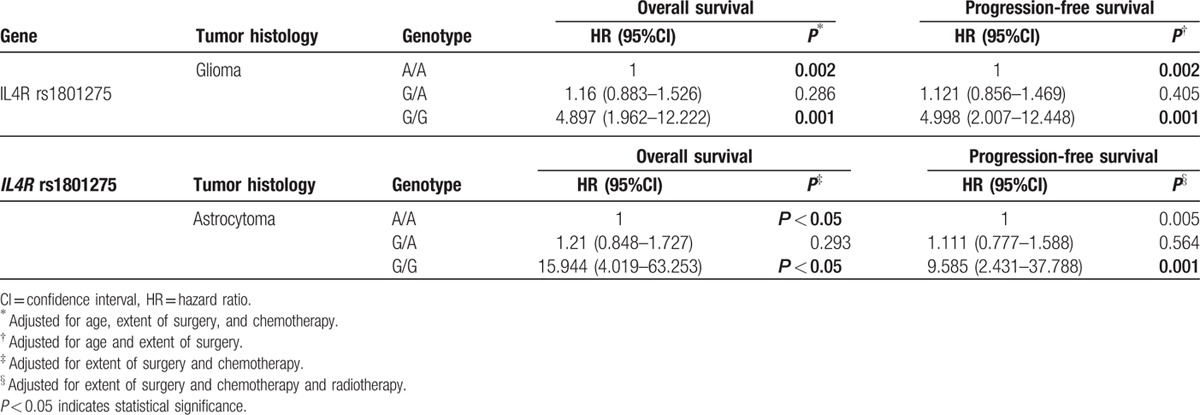
Associations between *IL4R* rs1801275 and glioma and astrocytoma.

## 4 Discussion

To our knowledge, this is the first analysis of *IL4R* gene in glioma patients from the Northwestern China. We showed that genetic variant GG of *IL4R* rs1801275 was significantly associated with poor survival of glioma and astrocytoma patients. What's more, we also found that the extent of surgical resection, chemotherapy, and age were key prognostic factors in glioma survival.

Surgery and chemotherapy are usually the initial therapy of choice in glioma treatment. In our research, we found that the extent of surgical resection and chemotherapy could influence the overall survival of patients. Patients receive surgery or chemotherapy had better prognosis than those without surgery or chemotherapy. One study with 205 patients who had different treatments found that age, KPS, location of tumor, radical surgery, chemotherapy, and radiotherapy all had significant effects on survival for patients with GBM. Survival time for radical surgery and partial surgery was 16 months and 8 months, respectively. Median survival for those who received radiotherapy was 15 months versus 8 months for patients who did not have such treatments.^[13]^ So, surgery and chemotherapy are the important factors influencing glioma prognosis.

Variants in inflammatory genes contribute to individual susceptibility in risk for atopic disorders, which have been linked to protection against various malignancies, including gliomas.^[14,15]^ Previous studies reported that rs1801275 in the *IL4R* gene could increase the risk of glioblastoma in a population-based case-control study.^[16,17]^ The *IL4R* gene affects both the risk of glioma and survival time. Results from previous study concerning that 2 nonsynonymous SNPs in the *IL4R* gene, rs1805015 and rs1805016, are significantly associated with long-term survival among high-grade glioma patients.^[9]^ In our study, we found that glioma patients with GG genotype had a poor survival. The 1-year overall survival of glioma patients with *IL4R* rs1801275 GG (0%) and GA (29.3%) were lower than the AA (31.5%) genotype. These observations suggest that *IL4R* mutations play a critical role in glioma pathogenesis and are believed to be linked with the progression of the disease.

*IL-4* is a key cytokine associated with allergy.^[18]^ It confers immunoregulatory activity by interacting with its cognate receptors, a complex of IL-4Rα with either the common γ-chain or the IL-13R chain α1.^[18,19]^*IL4R* encodes the alpha chain of the interleukin-4 receptor that can bind interleukin 4 and interleukin 13 to regulate IgE production.^[20]^ Hosoyama et al^[21]^ showed that IL-4R signaling pathway could modulate the progression of tumor cells, and it could be a promising therapeutic target for cancer by inhibiting this signaling pathway. Activation of the IL-4 pathway may lead to increased cell proliferation, cell growth, or apoptosis depending on which signal transduction pathway becomes initiated. Furthermore, certain *IL4R* SNPs, such as rs1805010, has already been shown to have a functional effect on the IgE level by upregulating the receptor's response to IL-4, which in turn results in activation of the Stat6 pathway.^[22]^ So, mutations in genes of *IL-4*, *IL-13*, and their receptors may break up the balance of cytokines network and contribute to allergy and carcinogenesis.

We found that extent of surgical resection, chemotherapy, and age is key prognostic factor in glioma survival. Moreover, to the best of our knowledge, the present study is the first time to investigate that *IL-4* rs1801275 mutations affect the progression of glioma patients. Collectively, these observations may be potentially valuable prognostic markers for glioma patients.
